# Evaluation of the *in vitro* and *in vivo* inhibitory effect of thymoquinone on piroplasm parasites

**DOI:** 10.1186/s13071-019-3296-z

**Published:** 2019-01-16

**Authors:** Shimaa Abd El-Salam El-Sayed, Mohamed Abdo Rizk, Naoaki Yokoyama, Ikuo Igarashi

**Affiliations:** 1 National Research Center for Protozoan Diseases, Obihiro University of Agriculture and Veterinary Medicine, Inada-Cho, Obihiro, Hokkaido Japan; 2 Department of Biochemistry and Chemistry of Nutrition, Faculty of Veterinary Medicine, Mansoura University, Mansoura, 35516 Egypt; 3 Department of Internal Medicine and Infectious Diseases, Faculty of Veterinary Medicine, Mansoura University, Mansoura, 35516 Egypt

**Keywords:** *Babesia*, *Theileria*, Thymoquinone, *In vitro*, *In vivo*

## Abstract

**Background:**

Developing new antibabesial drugs with a low toxic effect to the animal and with no resistance from *Babesia* parasites is in urgent demand. In this concern, the antimalarial, anticancer and antioxidant effect of thymoquinone (TQ), a phytochemical compound found in the plant *Nigella sativa*, has been reported. Therefore, in the present study, the antibabesial effect of this compound was evaluated on the growth of piroplasm parasites.

**Results:**

Significant inhibition (*P* < 0.05) of the *in vitro* growth of piroplasm parasites were observed after treatment by TQ with IC_50_ values of 35.41 ± 3.60, 7.35 ± 0.17, 0.28 ± 0.016, 74.05 ± 4.55 and 67.33 ± 0.94 μM for *Babesia bovis*, *Babesia bigemina*, *Babesia divergens*, *Theileria equi* and *Babesia caballi*, respectively. The *in vitro* inhibitory effect of TQ was significantly enhanced (*P* < 0.05) when used in combination with either diminazene aceturate on bovine *Babesia* and equine *Babesia* and *Theileria* cultures. In *B. microti*-infected mice, oral and intraperitoneal administrations of TQ showed significant (*P* < 0.05) inhibition of parasite growth at a dose of 70 mg/kg and 50 mg/kg, respectively, compared to the control group.

**Conclusions:**

The obtained results indicate that thymoquinone might be a promising medicinal compound for use in the treatment of animal piroplasmosis.

**Electronic supplementary material:**

The online version of this article (10.1186/s13071-019-3296-z) contains supplementary material, which is available to authorized users.

## Background

Piroplasmosis is a tick-borne disease of huge economic importance in the animal industry worldwide. The disease has zoonotic importance and is caused by hemoprotozoan parasites [1]. The main etiological agents of the disease in cattle are *Babesia bovis* and *B. bigemina* [1]. Moreover, *Babesia divergens* has a substantial impact on cattle health and productivity and has zoonotic importance [2]. In equines, the disease is caused by *Theileria equi* and *B. caballi* [3]. Generally speaking, babesiosis is typified in animals by high fever, hemolytic anemia and hemoglobinuria [1]. In fact, in recent years the inhibitory effects of many drugs such as epoxomicin, ciprofloxacin, thiostrepton and rifampicin, [4, 5] as well as pyronaridine tetraphosphate, luteolin, nimbolide, gedunin, enoxacin and N-acetyl-L-cysteine [6–8] have been evaluated against *Babesia* and *Theileria* parasites. However, none are yet available for commercial use in the veterinary market. Diminazene aceturate (DA) is commonly used to treat animal piroplasmosis under field conditions. However, it does not achieve complete elimination of parasites from hosts. Consequently, a relapse of infection in treated animals occurs [9]. Moreover, toxic side effects to the host and resistance from *Babesia* parasites has developed from the currently available antibabesial drugs in the veterinary field, imidocarb dipropionate and DA, respectively [9]. Therefore, developing new drugs with a low toxic effect to the animal and with no resistance from the parasite is a great priority. In this regard, *Nigella sativa* (black cumin) is one of the most important medicinal plants, known for its antioxidant, anti-inflammatory, antibacterial, antiviral, antiparasitic, anticarcinogenic, antiallergic, antidysenteric and antiulcer effects [10, 11]. Additionally, the immunoregulatory and immunomodulatory effects of this plant have been proven [11]. The main component within *N. sativa* is thymoquinone (TQ) (30–48%) [12]. Notably, the antimalarial efficacy of TQ has been proven *in vitro* and *in vivo* against the growth of *Plasmodium berghei* in mice [13]. Additionally, previous studies have proven the anticancer [14] and antioxidant [15] effects of TQ. Furthermore, this phytochemical compound can ameliorate the toxic effects of some conventional medicine such as cisplatin [16] and doxorubicin [17] on the kidney and heart, respectively. However, the antibabesial effect of TQ has not yet been evaluated. Therefore, in the present study, the potential of TQ against piroplasm parasites was evaluated.

## Methods

### Parasites

The Texas strain of *B. bovis*, [5, 6] the Argentina strain of *B. bigemina*, [6, 18] the German bovine strain of *B. divergens* [7, 8] and U.S. Department of Agriculture (USDA) strains of *B. caballi* [8] and *T. equi* [3, 6] were cultivated in purified bovine or equine red blood cells (RBCs) using a microaerophilic stationary-phase culture system [6, 18] and used for the *in vitro* experiment. The Munich strain of *B. microti* [4] was maintained by passage in the blood of BALB/c mice (CLEA, Tokyo, Japan) [19] and used in the *in vivo* study.

### Chemical reagents

TQ (Sigma-Aldrich, Tokyo, Japan) was prepared as 100 mM stock solutions and stored at -30 °C until use. Olive oil (Ajinomoto, Japan) was used for dissolving TQ. DA (Novartis, Tokyo, Japan) was used as a positive control drug. SYBR Green I (SGI) nucleic acid stain (Lonza, Rockland, USA; 10,000×) was mixed with a lysis buffer [Tris (130 mM; pH 7.5), EDTA (10 mM), saponin (0.016%; W/V) and TritonX-100 (1.6%; V/V)] and used for the fluorescence assay.

### TQ evaluation against *Babesia* and *Theileria* parasites *in vitro*

The inhibitory effects of TQ on piroplasma growth were evaluated using the *Babesia* fluorescence assay (BFA) previously described by Rizk et al. [6, 7]. Briefly, parasite-infected RBCs were cultivated in double 96-well plates (Nunc, Roskilde, Denmark) for 4 days using Medium 199 (for *B. bovis*, *B. bigemina* and *T. equi*) and RPMI 1640 medium (for *B. divergens* and *B. caballi*) (both from Sigma-Aldrich) alone or with the indicated concentrations: 0.25, 0.5, 1, 5 and 10 μM for DA or 0.01, 0.1, 0.25, 0.5, 1, 5, 10, 25, 50, 100, 200, 400 and 800 μM for TQ. Each drug concentration was loaded into each well in triplicate. On the fourth day of culture, 100 μl of lysis buffer was mixed with 2 × SGI and added to each well in the first 96-well plate for calculation the IC_50_ of TQ for each parasite. The second 96-well plate was used for evaluation of the parasites’ viability after treatment with TQ, as formerly described by Rizk et al. [7, 8]. The emitted fluorescence signals were determined using a fluorescence plate reader (Fluoroskan Ascent, Thermo Electron Informatics, Philadelphia, PA, USA).

### *In vitro* drug combination test

Different concentrations of TQ with the commonly used antibabesial drug, DA were applied as previously described [5, 8, 19, 20] with some modifications. Combinations of TQ and DA were examined against the *in vitro* culture of *B. bovis*, *B. bigemina*, *B. caballi* and *T. equi*. Untreated cultures and cultures treated with only DA IC_50_ for *Babesia* and *Theileria* parasites were used as controls. The fluorescence values were determined as described earlier [8] for the fluorescence assay.

### Chemotherapeutic evaluation of TQ in mice

The inhibitory effect of TQ against *B. microti* in a mouse model was performed in the present study using a fluorescence assay [21]. Collectively, 55 female 8-week-old BALB/c mice were used in the *in vivo* study. Thirty mice were used for evaluating the inhibitory effect of TQ on *B. microti* when administrated *via* the oral route, while another 25 mice were used for evaluating the antibabesial effect of intraperitoneal (IP) injection of TQ.

For the 30 mice that received TQ *via* the oral route, the mice were divided equally into six groups. Mice in the first five groups were intraperitoneally inoculated with 1 × 10^7^ *B. microti*-infected RBCs, while mice in the sixth group remained without infection and were used as a blank control. In the first and second groups, TQ was orally administered with non-toxic doses at a dose rate of 100 and 70 mg/kg, respectively [22]. Combination therapy consisting of 50 mg/kg TQ and 10 mg/kg DA was administrated in the same inoculation period to the mice in the third group by oral and subcutaneous routes, respectively. DA at a dosage of 25 mg/kg was subcutaneously administrated to the fourth experimental group. TQ and DA were dissolved in 0.1 ml olive oil or double distilled water (DDW), respectively. Olive oil was administered to mice in the fifth group as a placebo control.

The 25 mice that were treated with intraperitoneal (IP) injections of TQ were divided equally into five groups. Mice in the first four groups were intraperitoneally inoculated with *B. microti*-infected RBCs as described above, and mice in the fifth group remained without infection and were used as a blank control. Mice in the first and second groups received an intraperitoneal (IP) injection of TQ and subcutaneous (SC) injection of DA at dose rates of 25 and 50 mg/kg, respectively [22]. A combination of TQ (IP injection) and DA (SC injection) was administrated to mice in the third group at dose rates of 25 and 10 mg/kg, respectively, in the same inoculation period. Olive oil (0.1 ml) was administrated intraperitoneally to mice in the fourth group, and served as a positive control [22]. For all treated mice, the specific drug or solvent was administrated for 5 successive days when parasitemia reached 1% in the infected mice. Venous tail blood samples (2.5 μl) were collected from each mouse every 2 days until 20 days post-inoculation or the cessation of parasitemia. Non-parasitized RBCs from uninfected mice were used as a blank control. The blood samples were collected in a 96-well plate containing RPMI 1640 medium previously mixed with 50 μl of a lysis buffer to prevent blood coagulation. After that, 50 μl of a lysis buffer containing 2× SGI nucleic acid stain was added directly to each well and gently mixed. Next the plate was incubated in the dark for 1 h and the fluorescence values determined as described above using a fluorescence spectrophotometer.

### Role of TQ in treatment of anemia associated with *Babesia*

Venous tail blood samples (10 μl) were collected from all treated mice, and hematological variables including hematocrit (HCT) values, hemoglobin (HGB) levels, red blood cell (RBC) counts, mean corpuscular volume (MCV), mean corpuscular hemoglobin (MCH) and RBC distribution width (RDW) were measured and used as indicators of the anemia development in these mice. Mice that did not receive any infection or drug treatment served as a negative control. In all groups, the hematological parameters were monitored every 96 h using the Celltac α MEK-6450 automatic hematology analyzer (Nihon Kohden Corporation, Tokyo, Japan).

### Statistical analysis

The significant differences between groups were determined by independent Student’s t-test and one-way ANOVA test using GraphPad Prism version 5.0 for Windows (GraphPad Software Inc., San Diego, CA, USA). A *P*-value of < 0.05 was considered statistically significant. The regrowth of the parasite in the viability test was determined based on the statistically significant differences between drug-treated and positive control groups [7].

## Results

### *In vitro* inhibitory effect and drug combination test

The *in vitro* growth of *B. bovis* was significantly inhibited (t-test: *t*_(4)_ = 6.02, *P* = 0.003) by a 0.25 μM treatment of TQ. The *in vitro* growth of the other *Babesia* and *Theileria* parasites tested was significantly inhibited (t-test: *t*_(4)_ = 12.01, *P* = 0.0003 for *B. bigemina*; *t*_(4)_ = 54.46, *P* < 0.0001 for *B. divergens*; *t*_(4)_ = 6.47, *P* = 0.002 for *T. equi*; and *t*_(4)_ = 19.77, *P* < 0.0001 for *B. caballi*) by a 0.01 μM treatment of TQ (Additional file 1: Figure S1). *Babesia divergens* and *B. bigemina* were the most susceptible parasites to the *in vitro* inhibition effect of TQ, followed by *B. bovis* (Table 1). In contrast, equine *Babesia* and *Theileria* parasites exhibited *in vitro* resistance to the TQ inhibitory effect (Table 1). Consequently, the ability of TQ to inhibit the regrowth of *Babesia* and *Theileria* parasites after 4 days of *in vitro* treatment was assessed using a viability test. The results revealed no regrowth of the *B. bovis*, *B. bigemina*, *B. divergens*, *T. equi* or *B. caballi* parasites at 400, 100, 5, 200 or 10 μM treatments, respectively. Moreover, a 0.5 μM DA treatment was sufficient to suppress the regrowth of *Babesia* and *Theileria* parasites.

**Table 1:** IC_50_ values of thymoquinone and diminazene aceturate evaluated for bovine *Babesia* and equine *Babesia* and *Theileria* parasites

Organism	IC_50_ (μM)^a^	
	Thymoquinone	Diminazene aceturate
*B. bovis*	35.41 ± 3.60	0.415 ± 0.07
*B. bigemina*	7.35 ± 0.17	0.209 ± 0.005
*B. divergens*	0.28 ± 0.016	0.12 ± 0.03
*T. equi*	74.05 ± 4.55	0.709 ± 0.047
*B. caballi*	67.33 ± 0.94	0.007 ± 0.0002

^a^IC_50_ values for thymoquinone and diminazene aceturate were calculated on the fourth day based on the growth inhibitions determined using a fluorescence-based assay in three separate experiments. Each drug concentration was made in triplicate in each experiment, and the final obtained IC_50_ represents the mean and standard deviation of three separate experiments

Notably, combination therapy consisting of 4.44 μM TQ (1/16 IC_50_) and 0.35 μM DA (1/2 IC_50_) significantly enhanced the growth inhibition of *T. equi* better than those observed by DA IC_50_ (Table 2). Furthermore, 1.00 μM TQ (1/16 IC_50_) and 0.10 μM DA (1/2 IC_50_), 0.14 μM TQ (1/16 IC_50_) and 0.10 μM DA (1/2 IC_50_), and 9.38 μM TQ (1/16 IC_50_) and 0.0035 μM DA (1/2 IC_50_) significantly enhanced the inhibitory effect of TQ on *B. bovis* (Table 3), *B. bigemina* (Table 4) and *B. caballi* (Table 5), respectively, in *in vitro* cultures. Moreover, treatments of *B. bovis*, *B. bigemina* and *B. caballi* parasites with TQ and DA combination therapy enhanced their growth inhibition significantly better than those observed by DA IC_50_ for *B. bovis* (8.87 μM TQ (1/4 IC_50_): 0.30 μM DA (3/4 IC_50_) (Table 3), *B. bigemina* (1.83 μM TQ (1/4 IC_50_): 0.10 μM DA (1/2 IC_50_) (Table 4) and *B. caballi* ( 9.38 μM TQ (1/16 IC_50_): 0.0052 μM DA (3/4 IC_50_) (Table 5). These results confirmed the potential antibabesial effect of TQ, especially when administrated simultaneously with DA.

**Table 2:** Concentrations and growth inhibition effect of thymoquinone combined with diminazene aceturate applied to the cultures of *T. equi*

Concentration (μM)^a^		Fluorescence value^b^	t-test^c^			
			*t-*value		*P-*value	
TQ	DA		Control	DA IC_50_	Control	DA IC_50_
55.50	0.52	8.22 ± 0.51	51.44	374.20	<0.0001	<0.0001
55.50	0.35	9.67 ± 0.12	51.25	819.37	<0.0001	<0.0001
37:00	0.52	11.75 ± 0.77	50.73	251.90	<0.0001	<0.0001
37:00	0.35	11.32 ± 0.47	50.91	390.17	<0.0001	<0.0001
18.50	0.52	14.68 ± 0.42	50.33	414.77	<0.0001	<0.0001
18.50	0.35	16.31 ± 0.26	50.07	569.11	<0.0001	<0.0001
4.44	0.52	17.09 ± 0.14	49.94	738.13	<0.0001	<0.0001
4.44	0.35	19.21 ± 0.79	49.41	230.24	<0.0001	<0.0001
Control		299.71 ± 9.80				
DA IC_50_		128.22 ± 0.22				

^a^Combinations were based on the calculated IC_50_ values obtained from the *in vitro Babesia* fluorescence assay

^b^Each value was calculated using a fluorescence-based assay in three separate experiments. Each concentration of the drug combination was made in triplicate in each experiment, and the final obtained fluorescence values represent the mean and standard deviation (SD) of three separate experiments after subtracting the background fluorescence for non-parasitized RBCs

^c^t-test represents the statistically significant differences between the combined drug-treated group and either the control group or the diminazene aceturate-treated group

*Abbreviations*: *TQ* thymoquinone, *DA* diminazene aceturate

**Table 3:** Concentrations and growth inhibition effect of thymoquinone combined with diminazene aceturate applied to the cultures of *B. bovis*

Concentration (μM)^a^		Fluorescence value^b^	t-test^c^			
			*t-*value		*P-*value	
TQ	DA		Control	DA IC_50_	Control	DA IC_50_
26.62	0.30	13.79 ± 0.56	78.53	256.26	<0.0001	<0.0001
26.62	0.10	20.13 ± 0.58	76.42	235.60	<0.0001	<0.0001
17.75	0.30	87.01 ± 0.20	54.74	115.21	<0.0001	<0.0001
17.75	0.10	90.42 ± 0.51	53.40	73.90	<0.0001	<0.0001
8.87	0.30	111.99 ± 1.54	44.63	7.75	<0.0001	0.0015
8.87	0.10	163.91 ± 3.11	25.24	24.67	<0.0001	<0.0001
1.00	0.30	175.62 ± 1.88	23.99	50.64	<0.0001	<0.0001
1.00	0.10	183.29 ± 2.68	20.43	40.89	<0.0001	<0.0001
Control		252.74 ± 5.24				
DA IC_50_		119.16 ± 0.44				

^a^Combinations were based on the calculated IC_50_ values obtained from the *in vitro Babesia* fluorescence assay

^b^Each value was calculated using a fluorescence-based assay in three separate experiments. Each concentration of the drug combination was made in triplicate in each experiment, and the final obtained fluorescence values represent the mean and standard deviation (SD) of three separate experiments after subtracting the background fluorescence for non-parasitized RBCs

^c^t-test represents the statistically significant differences between the combined drug-treated group and either the control group or the diminazene aceturate-treated group

*Abbreviations*: *TQ* thymoquinone, *DA* diminazene aceturate

**Table 4:** Concentrations and growth inhibition effect of thymoquinone combined with diminazene aceturate applied to the cultures of *B. bigemina*

Concentration (μM)^a^		Fluorescence value^b^	t-test^c^			
			*t-*value		*P-*value	
TQ	DA		Control	DA IC_50_	Control	DA IC_50_
5.51	0.15	8.81 ± 1.03	287.14	59.50	<0.0001	<0.0001
5.51	0.10	9.13 ± 1.60	213.64	54.50	<0.0001	<0.0001
3.67	0.15	11.40 ± 0.97	293.43	58.36	<0.0001	<0.0001
3.67	0.10	12.09 ± 2.20	162.84	47.52	<0.0001	<0.0001
1.83	0.15	26.17 ± 2.88	120.20	35.62	<0.0001	<0.0001
1.83	0.10	33.46 ± 0.86	281.21	45.44	<0.0001	<0.0001
0.14	0.15	207.37 ±1.54	27.38	56.66	<0.0001	<0.0001
0.14	0.10	223.99 ± 2.27	8.21	58.04	0.0012	<0.0001
Control		235.57 ± 0.90				
DA IC_50_		106.81 ± 2.66				

^a^Combinations were based on the calculated IC_50_ values obtained from the *in vitro Babesia* fluorescence assay

^b^Each value was calculated using a fluorescence-based assay in three separate experiments. Each concentration of the drug combination was made in triplicate in each experiment, and the final obtained fluorescence values represent the mean and standard deviation (SD) of three separate experiments after subtracting the background fluorescence for non-parasitized RBCs

^c^t-test represents the statistically significant differences between the combined drug-treated group and either the control group or the diminazene aceturate-treated group

*Abbreviations*: *TQ* thymoquinone, *DA* diminazene aceturate

**Table 5:** Concentrations and growth inhibition effect of thymoquinone combined with diminazene aceturate applied to the cultures of *B. caballi*

Concentration (μM)^a^		Fluorescence value^b^	t-test^c^			
			*t-*value		*P-*value	
TQ	DA		Control	DA IC_50_	Control	DA IC_50_
50.25	0.0052	79.25 ± 0.47	450.67	156.58	<0.0001	<0.0001
50.25	0.0035	92.75 ± 1.45	216.27	54.18	<0.0001	<0.0001
33.50	0.0052	104.32 ±1.02	266.65	55.57	<0.0001	<0.0001
33.50	0.0035	113.25 ±1.70	169.62	26.80	<0.0001	<0.0001
16.75	0.0052	115.22 ± 0.83	287.37	45.66	<0.0001	<0.0001
16.75	0.0035	121.89 ± 0.71	302.17	37.62	<0.0001	<0.0001
9.38	0.0052	125.12 ± 0.85	267.38	27.38	<0.0001	<0.0001
9.38	0.0035	162.39 ± 2.60	83.74	14.24	<0.0001	0.0001
Control		292.20 ± 0.67				
DA IC_50_		140.63 ± 0.49				

^a^Combinations were based on the calculated IC_50_ values obtained from the *in vitro Babesia* fluorescence assay

^b^Each value was calculated using a fluorescence-based assay in three separate experiments. Each concentration of the drug combination was made in triplicate in each experiment, and the final obtained fluorescence values represent the mean and standard deviation (SD) of three separate experiments after subtracting the background fluorescence for non-parasitized RBCs

^c^t-test represents the statistically significant differences between the combined drug-treated group and either the control group or the diminazene aceturate-treated group

*Abbreviations*: *TQ* thymoquinone, *DA* diminazene aceturate

### *In vivo* effect of TQ on *B. microti* infection

**Fig. 1:**
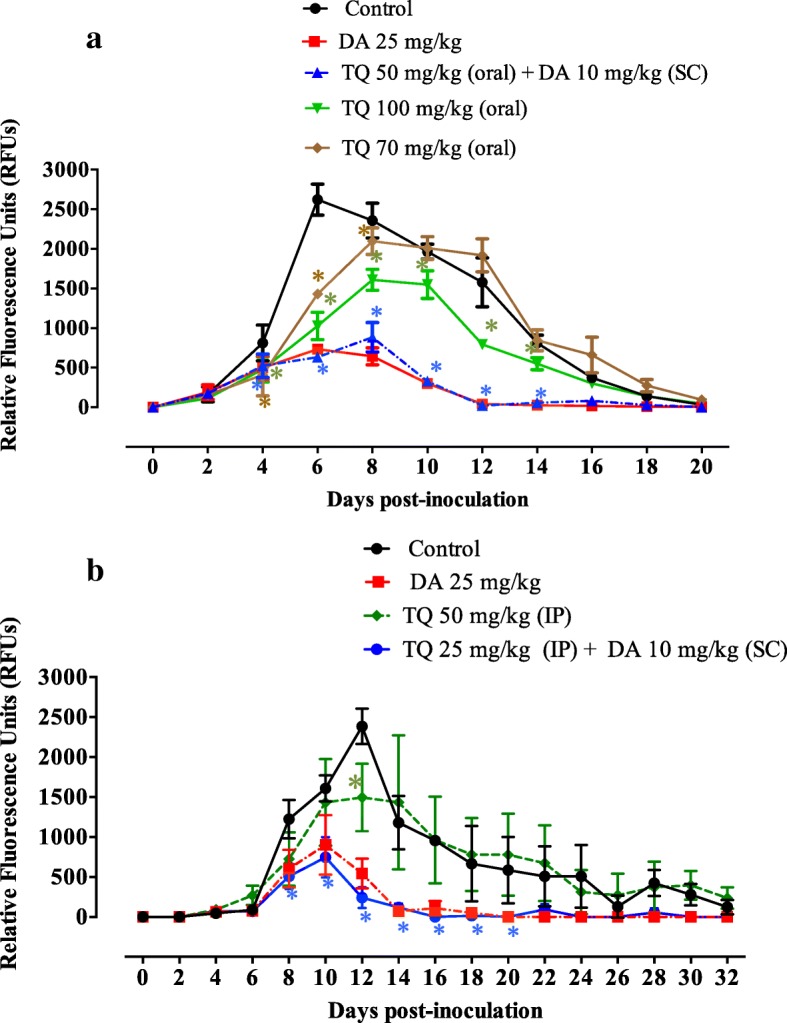
Inhibitory effect of diminazene aceturate, thymoquinone, and a combination therapy of both drugs on the growth of *Babesia microti*. **a** Oral administration of TQ. **b** Intraperitoneal administration of TQ. Each value represents the mean ± standard deviation of five mice per experimental group after subtracting the background fluorescence for non-parasitized RBCs from uninfected mice. Asterisks indicate significant differences (*P* < 0.05) between treated and control mice. Gain values are set to 100. *Abbreviations*: TQ, thymoquinone; DA, diminazene aceturate; SC, subcutaneous administration; IP, intraperitoneal administration

In the present study, TQ was administered *via* two routes: oral and intraperitoneal. In mice that received oral TQ treatment, fluorescence values were significantly inhibited (ANOVA: *F*_(1.442,14.42)_ = 8.84, *P* = 0.005) in the group treated with either TQ alone at a dose rate of 100 mg/kg or TQ in combination with DA from days 4 to 14 post-inoculation (pi) as compared to control mice (Fig. 1a). Treatment with TQ at a dose rate of 70 mg/kg resulted in significant inhibition of fluorescence values (ANOVA: *F*_(1.442,14.42)_ = 8.84, *P* = 0.005) from day 4 to 8 pi as compared to control mice. Control mice treated with olive oil and mice treated with DA alone showed peak fluorescence signals at 6 days pi. Mice treated with TQ alone or in combination with DA showed peak fluorescence signals at 8 days pi, indicating the potential effect of TQ in hindering parasitemia development (Fig. 1a). Notably, mice treated with a combined therapy exhibited inhibition in the fluorescence values, similar to those obtained from mice treated with 25 mg/kg DA (Fig. 1a). Oral administration of 100 mg/kg TQ caused 61 and 32% inhibition rates in the parasite growth at 6 and 8 days pi, respectively. Furthermore, the inhibition rates observed with 70 mg/kg oral doses of TQ were 45% at 6 days pi and 11% at 8 days pi (Fig. 1a). A 50 mg/kg oral dose of TQ combined with 10 mg/kg DA resulted in a 76% inhibition of *B. microti* growth at day 6 pi compared with 72% inhibition in the presence of 25 mg/kg DA at day 6 pi (Fig. 1a). However, at day 8 pi, TQ/DA combination therapy caused a 62.5% inhibition rate in parasite growth, and 25 mg/kg DA resulted in a 72.5% inhibition rate (Fig. 1a).

For the mice receiving IP treatment with TQ, the fluorescence values were significantly inhibited (ANOVA: *F*_(1.342,21.48)_ = 17.33, *P* = 0.0002) in mice treated with a TQ/DA combination from days 8 to 20 pi as compared to those in the control mice (Fig. 1b). Treatment with TQ at a dose rate of 50 mg/kg resulted in significant inhibition of fluorescence values (ANOVA: *F*_(1.342,21.48)_ = 17.33, *P* = 0.0002) at day 12 pi as compared to the control mice (Fig. 1b). Intraperitoneal treatment of *B. microti*-infected mice with 50 mg/kg TQ produced a 37% inhibition rate in parasite growth at 12 days pi (Fig. 1b). Control mice treated with olive oil and mice treated with TQ alone showed peak fluorescence signals at day 12 pi, while mice treated with TQ/DA combination therapy showed peak fluorescence signals at day 10 pi (Fig. 1b). Noticeably, mice IP injections from the TQ/DA combination formula exhibited fluorescence values lower than those obtained from mice treated with 25 mg/kg DA alone on the days of peak parasitemia (Fig. 1b). Intraperitoneal administration of TQ in combination with a subcutaneous dose of DA resulted in 54 and 90% inhibition rates of *B. microti* growth at 10 and 12 days pi, respectively (Fig. 1b). Interestingly, the inhibition rates caused by this combination therapy were higher than the 44 and 77% inhibition rates caused by DA alone at a dose rate of 25 mg/kg at days 10 and 12, respectively (Fig. 1b). Collectively, these results reveal that the TQ/DA combination seems to be more effective than treatment with TQ alone and highlights the fact that a combination formula of TQ and DA may be a promising treatment for babesiosis under field conditions.

### Role of TQ in retreating of anemia

**Fig. 2:**
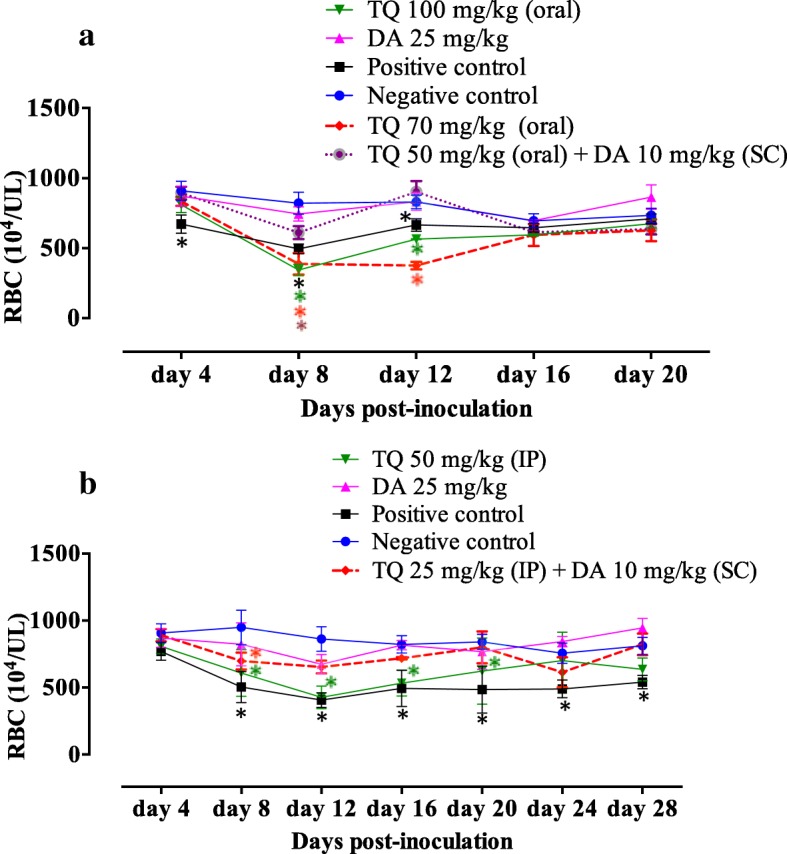
RBC counts in mice treated with thymoquinone. **a** Oral administration of TQ. **b** Intraperitoneal administration of TQ. Each value represents the mean ± standard deviation of five mice per experimental group. Asterisks indicate a significant difference (*P* < 0.05) between treated or infected mice and uninfected mice. *Abbreviations*: TQ, thymoquinone; DA, diminazene aceturate; SC, subcutaneous administration; IP, intraperitoneal administration

The role of TQ in mouse recovery from anemia was evaluated by monitoring the changes in hematological variables. For mice receiving an oral dose of TQ, a significant reduction (ANOVA: *F*_(2.143,8.572)_ = 5.15, *P* = 0.03) in RBC count was observed in *B. microti*-infected mice treated with olive oil (positive control, PC) and TQ alone on days 4, 8 and 12 pi compared to uninfected mice (Fig. 2a). Additionally, a significant reduction (ANOVA: *F*_(2.143,8.572)_ = 5.15, *P* = 0.03) in RBC count was observed in mice treated with TQ combined with DA compared to uninfected mice at 8 days pi (Fig. 2a). A significant reduction (ANOVA: *F*_(2.295,9.178)_ = 6.47, *P* = 0.01) in HGB level was observed in mice treated with olive oil alone (PC) at 4, 8 and 12 days pi (Additional file 2: Figure S2a). In the same way, such a reduction was perceived in groups treated with TQ alone at days 12 and 16 pi. Unfortunately, mice receiving an oral dose of TQ alone or olive oil showed a significant reduction (ANOVA: *F*_(1.484,5.936)_ = 8.90, *P* = 0.01) in HCT levels until day 20 pi (Additional file 2: Figure S2b). Surprisingly, no such reduction was observed in HGB and HCT levels in mice treated with TQ combined with DA (Additional file 2: Figure S2a, b). Macrocytic anemia and anisocytosis were observed in all treated mice from day 8 pi, while from day 12 pi, combination therapy with TQ and DA had the ability to eliminate this anemia and yield a uniform population of red blood cells (Additional file 2: Figure S2c, e). Subsequently, a significant increase (ANOVA: *F*_(1.734,6.935)_ = 5.64, *P* = 0.03) in the MCH level was observed in mice that received oral doses of TQ or olive oil alone from day 8 pi (Additional file 2: Figure S2d).

Notably, intraperitoneal administration of TQ at 25 mg/kg in combination with 10 mg/kg DA was sufficient to normalize the RBC count (Fig. 2a), HGB level and HCT percentage at day 12 pi in treated mice (Additional file 3: Figure S3a, b). In contrast, the significant reduction in RBC count (ANOVA: *F*_(2.817,16.90)_ = 25.87, *P* < 0.0001), HGB level (ANOVA: *F*_(3.098,18.59)_ = 9.35, *P* = 0.0005) and HCT percentage (ANOVA: *F*_(2.661,15.97)_ = 12.90, *P* = 0.0002) was still detected in *B. microti*-infected mice treated with intraperitoneal doses of TQ alone (50 mg/kg) on days 20, 12 and 12 pi, respectively (Fig. 2a and Additional file 3: Figure S3a, b). All treated mice exhibited a significant elevation in MCV (ANOVA: *F*_(2.068,12.41)_ = 19.01, *P* = 0.0002) and RDW (ANOVA: *F*_(1.312,7.874)_ = 7.21, *P* = 0.0231) levels at day 12 pi, indicating the presence of macrocytic anemia and anisocytosis (Additional file 3: Figure S3c, e). Subsequently, a significant increase (ANOVA: *F*_(2.203,13.22)_ = 10.56, *P* = 0.0015) in the MCH level was observed in mice that received intraperitoneal doses of TQ (50 mg/kg) or olive oil alone from day 12 pi (Additional file 3: Figure S3d). Interestingly, at day 16 pi, this anemia and disturbances in the RBC population disappeared in mice treated with intraperitoneal doses of DA (25 mg/kg) alone or DA (10 mg/kg) in combination with TQ (25 mg/kg) (Additional file 3: Figure S3c). These findings highlight the efficacy of low oral or intraperitoneal doses of TQ and DA in combination with a fast recovery from anemia and anisocytosis in treated mice and indicate the potential antibabesial effect of this combination therapy.

## Discussion

Thymoquinone is an ancient phytochemical compound and its wide pharmacological activity as a hepatoprotective, cardioprotective, anti-inflammatory, anti-convulsant, anti-microbial, anti-histaminic and anti-diabetic has been evidently proved [23, 24]. Additionally, the therapeutic effect of TQ in treating many clinical disorders such as acute gastric ulcer, neurodegenerative and neuropsychiatric diseases, rheumatoid arthritis, and respiratory disorders including asthma and dyspnea has been reported [24]. In this regard, the antibabesial effect of TQ has been evaluated in the present study.

The IC_50_ values of TQ for piroplasma parasites were higher than those of pyronaridine tetraphosphate, luteolin, nimbolide and gedunin [6, 7]; MMV006706, MMV666093, MMV396693, MMV073843 and MMV665875 hits [25]; clofazimine [26]; epoxomicin [5]; ciprofloxacin, thiostrepton, and rifampicin [4]; DA and imidocarb dipropionate (commonly used antibabesial drugs) [20]; enoxacin against *B. bovis*, *T. equi* and *B. caballi* [6]; and fusidic acid and clindamycin phosphate against equine *Babesia* and *Theileria* parasites [27, 28]. On the other hand, the IC_50_ values of TQ for piroplasma parasites were lower than those of N-Acetyl-L-cysteine [8], allicin [19], fusidic acid, clodinafop-propargyl, and clindamycin phosphate against bovine *Babesia* parasites [20, 28]; chloroquine diphosphate and metronidazole against *B. gibsoni* [27]; and enoxacin against *B. bigemina* [6]. The viability test results exhibited the ability of TQ to suppress the regrowth of piroplasma parasites at lower concentrations in comparison with the results observed for allicin [19] and N-Acetyl-L-cysteine [8] on *B. bigemina*, *B. divergens*, *T. equi* and *B. caballi*, and for fusidic acid on *B. caballi* [28]. The *in vitro* application of TQ inhibited regrowth of the *B. bovis* parasite at concentrations similar to those observed for N-Acetyl-L-cysteine [8] and fusidic acid [28] on *B. bovis*, *B. bigemina* and *T. equi*.

To further validate the antibabesial efficacy of TQ, the *in vivo* inhibitory effect of TQ was evaluated in *B. microti*-infected mice in two separate trials. Even the same drug showed different chemotherapeutic effects with different administrative routes [29]. A recent study [26] in our laboratory reported different inhibitory effects of clofazimine on the growth of *B. microti* in mice when administrated in two different routes. Oral administration of the drug showed a much higher effect than the intraperitoneal route. Therefore, in the present study, to evaluate the possible effect of different administration methods of TQ on the growth of the *Babesia* parasite, TQ was administrated *via* oral and intraperitoneal routes. Intraperitoneal treatment of *B. microti*-infected mice with 50 mg/kg TQ produced a higher inhibition rate (37%) than those of (11 and 32% for 70 and 100 mg/kg TQ, respectively) by oral TQ treatment on the days of peak parasitemia. Furthermore, the inhibition rates in the fluorescence values obtained from treatment of *B. microti*-infected mice with TQ/DA combination therapy were higher in mice which received intraperitoneal injections of TQ than in mice treated orally with TQ when compared with the inhibition rates caused by DA alone at a dose rate of 25 mg/kg on the days of peak parasitemia. Generally speaking, the rate of absorption is the fastest for the intravenous route and then decreases in the following order: intraperitoneal > intramuscular > subcutaneous > oral [30]. Therefore, for the present study, the intraperitoneal administration of TQ seems to be more effective than oral administration for the treatment of *Babesia* parasites.

The *in vivo* inhibitory effects of TQ against *B. microti* when administrated *via* the oral route are lower than 77.5% inhibition for 500 mg/kg thiostrepton, 68.5% for 500 mg/kg clindamycin, 84% for 10 mg/kg (-)-epigallocatechin-3-gallate, 86% for 2.5 mg/kg heparin, and 72.4% for 100 mg/kg allicin [18, 19, 31]. In contrast, the inhibition rate of *B. microti in vivo* growth caused by a 100 mg/kg oral dose of TQ was higher than 53.7% for 100 mg/kg nerolidol, 58.3% for 30 mg/kg allicin, and 36.3 and 47.6% for 0.05 and 0.5 mg/kg epoxomicin, respectively [5, 19, 32]. Combination therapy of TQ with lower doses of DA was tested in mice infected with *B. microti*. The *in vivo* inhibitory effects of TQ when administrated *via* the oral route in combination with a subcutaneous doses of DA against *B. microti* are lower than 88.3, 87.1, 89.9 and 91.9% inhibition from 12.5 mg/kg DA combined with 15 mg/kg allicin and 6.75 mg/kg DA combined with 15, 30 or 50 mg/kg allicin, respectively [19]. On the other hand, the inhibition rate caused by oral TQ and DA combination was higher than that caused by clindamycin combined with the natural product quinine, which showed 70% inhibition of the growth of *B. microti* on day 7 pi [33].

The inhibition rate caused by intraperitoneal treatment of 50 mg/kg TQ against *B. microti*-infected mice are lower than 77.5% inhibition for 500 mg/kg thiostrepton, 68.5% for 500 mg/kg clindamycin, 84% for 10 mg/kg (−)-epigallocatechin-3-gallate, 86% for 2.5 mg/kg heparin, 72.4% for 100 mg/kg allicin, 53.7% for 100 mg/kg nerolidol, and 58.3% for 30 mg/kg allicin, respectively [4, 18, 19, 31]. In contrast, the inhibition rate of *B. microti in vivo* growth caused by a 50 mg/kg IP dose of TQ was higher than 36.3% for 0.05 mg/kg epoxomicin [5].

The inhibition rate of intraperitoneal administration of TQ in combination with a subcutaneous dose of DA against *B. microti* are similar to the 88.3, 87.1, 89.9 and 91.9% inhibition for 12.5 mg/kg DA combined with 15 mg/kg allicin and 6.75 mg/kg DA combined with 15, 30 or 50 mg/kg allicin, respectively [19]. In contrast, the inhibition rate caused by the intraperitoneally administered dose of TQ combined with the SC-administered dose of DA was higher than that caused by clindamycin combined with the natural product quinine, which showed a 70% inhibition of the growth of *B. microti* on day 7 pi [33].

Interestingly, the mice treated with combination therapy consisting of low oral or intraperitoneal doses of TQ and DA in combination exhibited a quick recovery from anemia and anisocytosis associated with babesiosis. Such findings are similar to those caused by enoxacin combined with DA in *B. microti*-infected mice [34]. The significant reductions in hematological variables observed in olive oil-treated control mice were similar to those previously detected in eight-week-old BALB/c mice infected with *B. microti* [26].

The results obtained in the present study and the long history of the successful application of TQ in treating different diseases makes TQ a promising candidate for drug development. However, all of the previous studies were performed in experimental animal models which neglected clinical evaluation of TQ efficacy, either in animal or human subjects. In fact, the clinical trials and pharmaceutical development of any promising drug candidate to formulate it into the conventional dosage forms such as tablet and capsule is necessary for translation the experimental findings into reality. Unfortunately, TQ is a phytochemical which has potent hydrophobicity, lipophilicity and a highly thermolabile nature, thereby hindering pharmaceutical development for use in clinical trials [24]. In this regard, many strategies in the last few years have been developed for the pharmaceutical formulation of TQ in order to improve its bioavailability without compromising efficacy and safety, such as construction of TQ using novel nanoformulations or polymeric micelle [24].

It should be noted that there are some limitations to the present study. Although a TQ and DA combination exhibited a potential antibabesial effect, the reason behind this potential inhibitory effect in mice infected with *B. microti* is not fully understood. Therefore, further studies are required to clarify this relationship. Additional studies are also warranted to analyze the possible synergistic effect of TQ and other recently identified potent antibabesial drugs, such as clofazimine [26] and pyronaridine tetraphosphate, [6, 7] when administered in combination with each other. Such analysis will help to determine the most effective composition ratio for treatment of *Babesia* and *Theileria* in animals in clinical applications.

## Conclusions

In the present study, the inhibitory effects of TQ were demonstrated against piroplasma parasites *in vitro* and *in vivo*. The results revealed that TQ exhibited the highest activity against the *in vitro* growth of *B. divergens* and *B. bigemina*. Notably, mice treated with a combined therapy with lower doses of TQ and DA exhibited a significant reduction in fluorescence values similar to those obtained from mice treated with high doses of a commonly used antibabesial drug (DA). These results indicate that TQ might be used for the treatment of animal piroplasmosis under field conditions.

## Additional files

Additional file 1: **Figure S1.** Correlation between RFUs and the log-concentrations of thymoquinone (nM) on *Babesia* and *Theileria* parasites. Each value represents the mean of triplicate experiments after subtracting the background fluorescence for non-parasitized RBCs. Gain values were set to 100. (PDF 98 kb)

Additional file 2: **Figure S2.** Anemia monitoring in mice treated with oral doses of thymoquinone. **a** Hemoglobin (HGB). **b** Hematocrit (HCT). **c** Mean corpuscular volume (MCV). **d** Mean corpuscular hemoglobin (MCH). **e** Red blood cell distribution width (RDW). Each value represents the mean ± standard deviation of five mice per experimental group. Asterisks indicate a significant difference (*P* < 0.05) between treated or infected mice and uninfected mice. *Abbreviations*: TQ, thymoquinone; DA, diminazene aceturate; SC, subcutaneous administration. (PDF 247 kb)

Additional file 3: **Figure S3.** Anemia monitoring in mice treated with intraperitoneal doses of thymoquinone. **a** Hemoglobin (HGB). **b** Hematocrit (HCT). **c** Mean corpuscular volume (MCV). **d** Mean corpuscular hemoglobin (MCH). **e** Red blood cell distribution width (RDW). Each value represents the mean ± standard deviation of five mice per experimental group. Asterisks indicate a significant difference (*P* < 0.05) between treated or infected mice and uninfected mice. *Abbreviations*: TQ, thymoquinone; DA, diminazene aceturate; SC, subcutaneous administration; IP, intraperitoneal administration. (PDF 265 kb)

## Abbreviations

DA: Diminazene aceturate
HCT: Hematocrit
HGB: Hemoglobin
IP: Intraperitoneal administration
MCH: Mean corpuscular hemoglobin
MCV: Mean corpuscular volume
RBC: Red blood cell
RDW: RBC distribution width
SC: Subcutaneous administration
SGI: SYBR Green I
TQ: Thymoquinone
