# Supplementary figures and images for: Evaluation of the in vitro and in vivo inhibitory effect of thymoquinone on piroplasm parasites
Source: Parasit Vectors. 2019 Jan 16;12:37. doi: 10.1186/s13071-019-3296-z (PMC6335684; doi:10.1186/s13071-019-3296-z)

Relative Fluorescence Units (RFUs)

200  
150  
100  
50  
0

0 1 2 3 4 5 6

log (nM)

- *B. bovis*
- *B. bigemina*
- ▲ *B. divergens*
- ▼ *T. equi*
- ◆ *B. caballi*

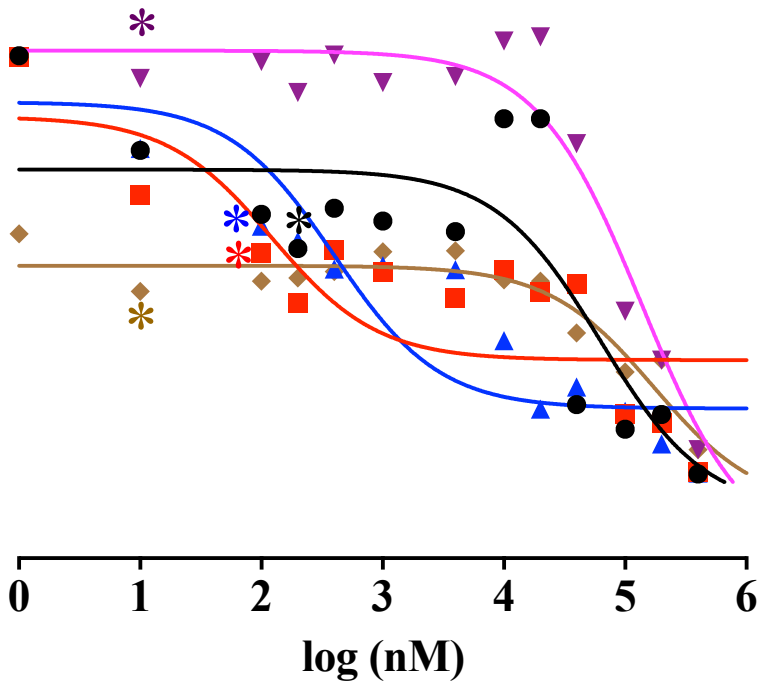

Supplement: Supplementary file 1 — Figure S1. Correlation between RFUs and the log-concentrations of thymoquinone (nM) on Babesia and Theileria parasites. Each value represents the mean of triplicate experiments after subtracting the background fluorescence for non-parasitized RBCs. Gain values were set to 100. (PDF 98 kb) [file 13071_2019_3296_MOESM1_ESM.pdf]

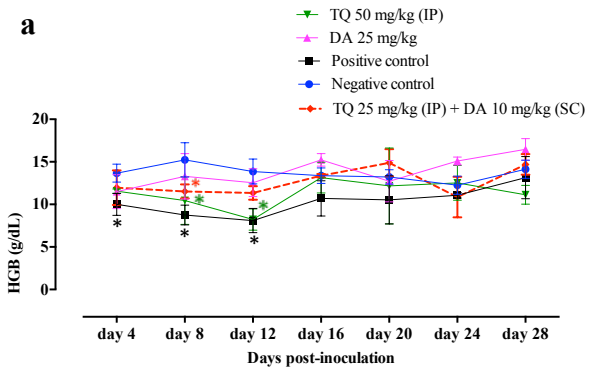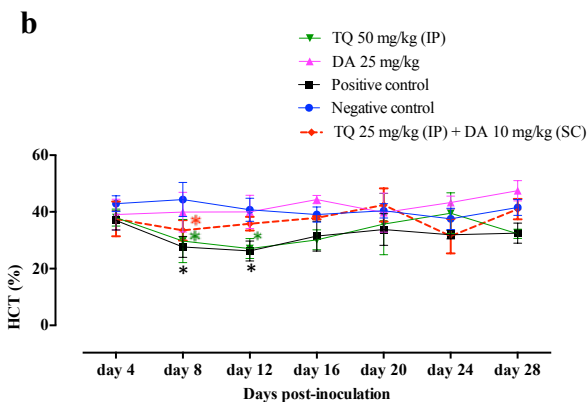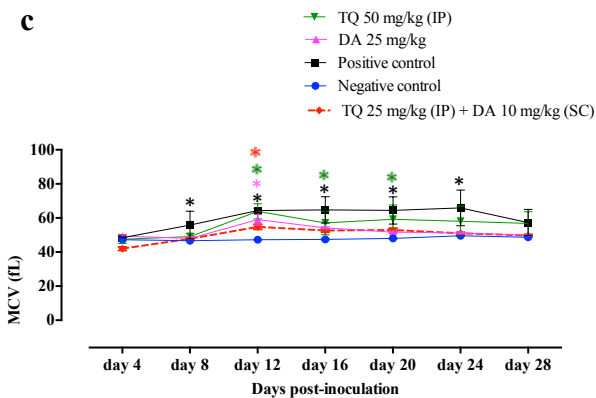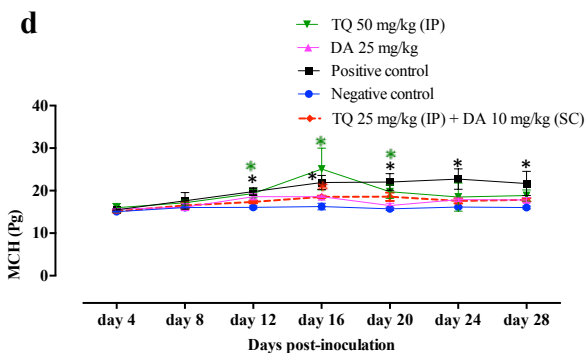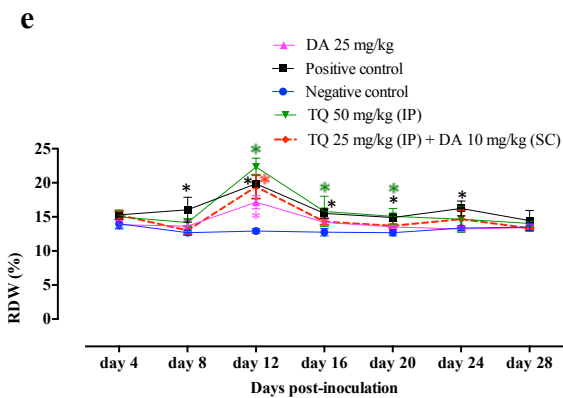

Supplement: Supplementary file 3 — Figure S3. Anemia monitoring in mice treated with intraperitoneal doses of thymoquinone. a Hemoglobin (HGB). b Hematocrit (HCT). c Mean corpuscular volume (MCV). d Mean corpuscular hemoglobin (MCH). e Red blood cell distribution width (RDW). Each value represents the mean ± standard deviation of five mice per experimental group. Asterisks indicate a significant difference (P < 0.05) between treated or infected mice and uninfected mice. Abbreviations: TQ, thymoquinone; DA, diminazene aceturate; SC, subcutaneous administration; IP, intraperitoneal administration. (PDF 265 kb) [file 13071_2019_3296_MOESM3_ESM.pdf]
