# Supplementary material for: Evaluation of the in vitro and in vivo inhibitory effect of thymoquinone on piroplasm parasites
Source: Parasit Vectors. 2019 Jan 16;12:37. doi: 10.1186/s13071-019-3296-z (PMC6335684; doi:10.1186/s13071-019-3296-z)
Supplement: Supplementary file 2 — Figure S2. Anemia monitoring in mice treated with oral doses of thymoquinone. a Hemoglobin (HGB). b Hematocrit (HCT). c Mean corpuscular volume (MCV). d Mean corpuscular hemoglobin (MCH). e Red blood cell distribution width (RDW). Each value represents the mean ± standard deviation of five mice per experimental group. Asterisks indicate a significant difference (P < 0.05) between treated or infected mice and uninfected mice. Abbreviations: TQ, thymoquinone; DA, diminazene aceturate; SC, subcutaneous administration. (PDF 247 kb) [file 13071_2019_3296_MOESM2_ESM.pdf]

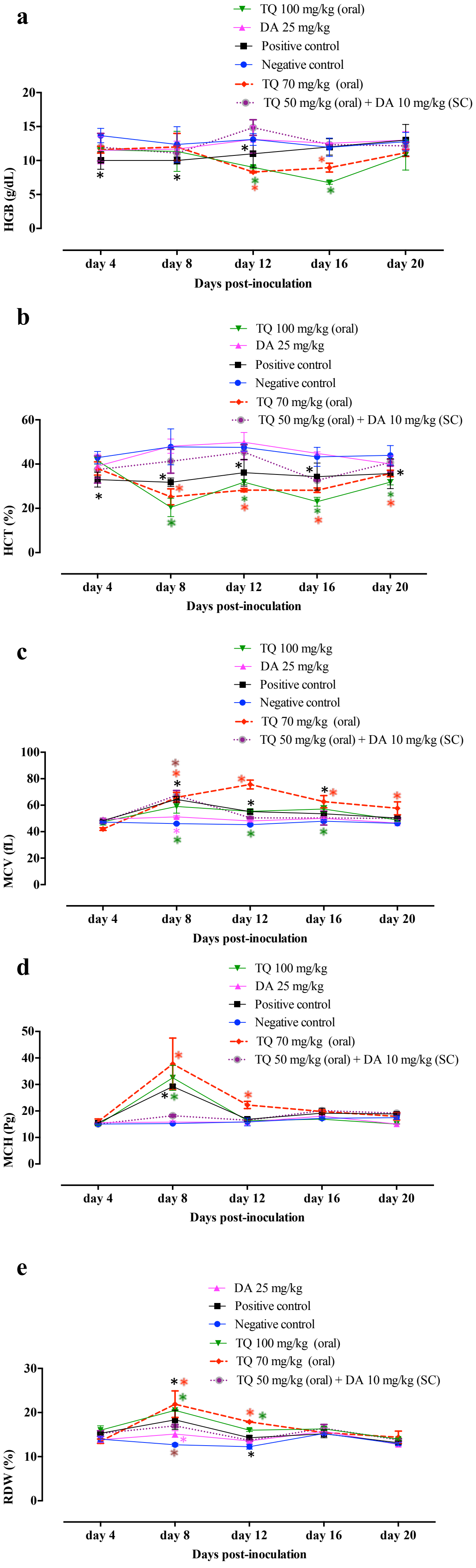
a
TQ 100 mg/kg (oral)
DA 25 mg/kg
Positive control
Negative control
TQ 70 mg/kg (oral)
TQ 50 mg/kg (oral) + DA 10 mg/kg (SC)
HGB (g/dL)
day 4
day 8
day 12
day 16
day 20
Days post-inoculation
b
TQ 100 mg/kg (oral)
DA 25 mg/kg
Positive control
Negative control
TQ 70 mg/kg (oral)
TQ 50 mg/kg (oral) + DA 10 mg/kg (SC)
HCT (%)
day 4
day 8
day 12
day 16
day 20
Days post-inoculation
c
TQ 100 mg/kg
DA 25 mg/kg
Positive control
Negative control
TQ 70 mg/kg (oral)
TQ 50 mg/kg (oral) + DA 10 mg/kg (SC)
MCV (fL)
day 4
day 8
day 12
day 16
day 20
Days post-inoculation
d
TQ 100 mg/kg
DA 25 mg/kg
Positive control
Negative control
TQ 70 mg/kg (oral)
TQ 50 mg/kg (oral) + DA 10 mg/kg (SC)
MCH (Pg)
day 4
day 8
day 12
day 16
day 20
Days post-inoculation
e
DA 25 mg/kg
Positive control
Negative control
TQ 100 mg/kg (oral)
TQ 70 mg/kg (oral)
TQ 50 mg/kg (oral) + DA 10 mg/kg (SC)
RDW (%)
day 4
day 8
day 12
day 16
day 20
Days post-inoculation
